# Dendritic Cells and Antiviral Defense

**DOI:** 10.3390/v12101152

**Published:** 2020-10-12

**Authors:** Robbie B. Mailliard

**Affiliations:** Department of Infectious Diseases and Microbiology, Graduate School of Public Health, University of Pittsburgh, Pittsburgh, PA 15261, USA; rbm19@pitt.edu

Dendritic cells (DCs) play a fundamental and central role in the immune response, acting as a critical link between the innate and adaptive branches of immunity. In the periphery, they become activated in response to ‘danger’ signals provided directly by microbial invaders or by other cells in the affected tissue microenvironment [1]. It is from here that they capture antigen and then migrate to draining lymphoid tissues to subsequently translate the acquired pathogen and tissue-derived information into the induction of appropriate adaptive T and B cell-mediated immune responses [2]. Due to their unique ability to effectively orchestrate primary adaptive immune responses, they have been widely explored and targeted for their therapeutic potential [1,3,4]. It is therefore not surprising that pathogens such as viruses have evolved to avoid, manipulate, or even utilize these central immune players to their advantage. In the special issue of Viruses entitled “Dendritic Cells and Antiviral Defense”, the functional role DCs play in anti-viral immunity and their therapeutic application was addressed by different research groups who contributed either reviews or primary research articles on the subject matter, which are summarized here.

In a review from the group of Jung et al. [5], the collective roles of different subsets of lung-associated DCs in protective immunity to respiratory syncytial virus (RSV) is examined. The authors detail the various pattern recognition receptors (PRRs) utilized by conventional DC1, DC2 and plasmacytoid DC (pDC) subsets to recognize and respond to RSV, including Toll-like receptors, retinoic acid-inducible gene-1 (RIG-1)-like receptors (RLRs), and nucleotide-biding oligomerization domain (NOD)-like receptors (NLRs), all of which are critical to controlling the virus. They also highlight mechanisms used by the virus to evade host immunity by interfering with DC function through the activity of the RSV proteins G, NS1, NS2, and N.

The role of DCs in immunity to the γ-herpesviruses Epstein Barr virus (EBV) and Kaposi sarcoma-associated herpesvirus (KSHV) is presented in a review by Christian Munz [6]. Although they are most often controlled by host immunity, complications associated with these viruses, such as the development of certain cancers, can occur during states of immunosuppression or with human immunodeficiency virus (HIV) co-infection. Munz describes preclinical models in which DCs are being a targeted as a strategic approach to induce γ-herpesvirus specific effector T cells capable of controlling these viruses as well as their associated cancers.

Virus exploitation of DC function is highlighted in a review from Perez-Zsolt et al., where the authors discuss how enveloped viruses, including HIV-1 and Ebola virus, incorporate sialic acid gangliosides on their membrane to facilitate viral attachment to the surface of activated DCs and DC-derived exosomes expressing Siglec-1 [7]. While the DC capture of viruses through this mechanism can promote antiviral immune responses, the authors explain how these viruses utilize this process to evade host immunity by negatively impacting DC function and to promote viral trafficking and dissemination.

A review by Ko et al. illustrates various ways in which DCs have been employed in the development of prophylactic and therapeutic HIV/SIV vaccines [8]. This report nicely details the historical use of DC-based cellular vaccines as well as other therapeutic strategies designed to target DC activity in vivo, covering the differential use of adjuvants, naonoparticles, nanopolymers, and viral mRNA-based approaches. In a related review from Kristoff et al. [9], the authors discuss the therapeutic potential for using type 1-polarized monocyte derived DCs (MDC1) to both induce HIV-1 specific CTL responses as well as to specifically activate and expose the HIV infected CD4+ T cells for immune attack in an all-in-one ‘kick and kill’ approach. Here, the authors propose the strategic use of both HIV- and CMV-associated MHC-Class II antigen in the design of a DC-based cellular vaccine, with the objective to target the HIV reservoir by exploiting the antigen specificity of those CD4+ T cells harboring latent, replication competent HIV proviral DNA.

The special issue was rounded off with two informative primary research studies. A report from Dewald et al. describes how pDC production of the antiviral factor interferon alpha (IFNα) by is tightly controlled through monocyte production of TNFα and its regulatory impact on the pDC transcription factor E2-2 [10]. In another study, Grosche et al. examines the negative impact of HSV-2 on DC function, describing the mechanism by which the virus triggers β2 integrin (LFA-1)-mediated adhesion events during the early stages of infection to impair the lymph node homing capacity of migratory DCs as a mechanism to hamper the immune response [11].

Together, these articles published in this special issue “Dendritic Cells and Antiviral Defense” provide a rich source of knowledge for those interested in the role of DCs in the host immune response to viral pathogens and the mechanisms exploited by viruses to subvert their powerful immune function. Those of us at the journal Viruses would like to thank those who contributed to this special issue, and we hope that the readers find this material useful and informative.
